# The Effect of Lipids on the Structure and Function of Egg Proteins in Response to Pasteurization

**DOI:** 10.3390/foods14020219

**Published:** 2025-01-13

**Authors:** Hao Yang, Qiang Shi, Zhongliang Wang, Xiao Chen, Fangfang Min, Xuanyi Meng, Ping Tong, Yong Wu, Hongbing Chen

**Affiliations:** 1 State Key Laboratory of Food Science and Resources, Nanchang University, Nanchang 330047, China; 352313318003@email.ncu.edu.cn (H.Y.); ncushiqiang@gmail.com (Q.S.); wangzhongl719@email.ncu.edu.cn (Z.W.); chenxiao6141@email.ncu.edu.cn (X.C.); min2fang@email.ncu.edu.cn (F.M.); mengxuanyi@ncu.edu.cn (X.M.); tongping@ncu.edu.cn (P.T.); ericyo918@hotmail.com (Y.W.); 2 Jiangxi Province Engineering Research Center of Special Medical Purposes Intended for Allergic Population, Nanchang University, Nanchang 330047, China; 3 College of Food Science and Technology, Nanchang University, Nanchang 330031, China; 4 Sino German Joint Research Institute, Nanchang University, Nanchang 330047, China

**Keywords:** liquid egg, protein–lipid interaction, protein structure, functional properties

## Abstract

In recent years, the consumption of liquid eggs has failed to meet the expectations of the public due to growing concerns regarding food safety and health. It is well known that there are interactions between the components in liquid eggs, and the interaction effect on the structure and functional properties of the proteins and antigenicity remains unclear. To investigate egg component interactions, we focused on four major egg lipids, namely phosphatidylcholine, palmitic acid, oleic acid, and linoleic acid, as well as four major egg proteins, including ovalbumin, ovotransferrin, ovomucoid, and lysozyme. The protein structural changes were analyzed using polypropylene gel electrophoresis, circular dichroism, ultraviolet absorption spectra, and exogenous fluorescence spectra, and the functional properties were assessed through solubility measurements and particle size analysis, while protein antigenicity was evaluated using an enzyme-linked immunosorbent assay. All the results revealed that oleic acid had the most significant effect on proteins’ secondary and tertiary structures, particularly affecting ovalbumin and ovotransferrin. Linoleic acid substantially increased the solubility of ovalbumin and ovomucoid, while palmitic acid significantly influenced the particle size of ovalbumin and lysozyme. Thus, we found that different lipids exhibit distinct effects on egg protein properties during pasteurization conditions, with oleic acid showing the most substantial impact on protein structure and antigenicity.

## 1. Introduction

Hens’ eggs represent a globally significant source of high-quality protein, containing all the essential amino acids required for human nutrition [1]. An egg consists of two primary components, egg white and egg yolk, each with distinct compositional characteristics. The protein content in egg whites ranges from 9.93% to 10.71% [2], contributing to crucial functional properties such as foaming and gelation [3]. Egg yolk contains 22.9% to 34.0% lipids, primarily comprising triglycerides (62.3%), phospholipids (32.8%), and sterols (cholesterol 5.0%), which are responsible for its emulsifying properties [2,4].

The major egg proteins that contribute to human nutrition include ovalbumin (OVA, 54%), ovotransferrin (OVT, 12%), ovomucoid (OVM, 11%), and lysozyme (LYZ, 3.5%) [5]. OVA, a 45 kDa (Dalton) protein, is extensively utilized in the food industry due to its diverse functional properties and serves as a model protein for physicochemical studies [6]. OVT, a 76 kDa iron-binding monomeric glycoprotein, plays a crucial role in egg albumin bioactivities [7]. OVM functions as a trypsin inhibitor, exhibiting remarkable thermal and anti-enzymatic stability [8]. LYZ, recognized as a natural antimicrobial agent, finds widespread application in food preservation techniques [9].

In egg yolk, phosphatidylcholine (PC) serves as an essential source of choline, benefiting human health [10] while providing superior emulsifying properties [11]. Palmitic acid (PA), a common saturated fatty acid, is abundant in both animal and plant sources [12]. Oleic acid (OA), an 18-carbon monounsaturated omega-9 fatty acid [13], is known to interact with protein molecules [14]. Linoleic acid (LA), an essential fatty acid, cannot be synthesized by mammals, including humans [15].

The fragility and perishability of eggshells present significant challenges in the food industry, as egg quality can deteriorate substantially over time. Consequently, liquid eggs, prepared by removing the shell, have gained increasing popularity [16]. These products are categorized as liquid egg white, liquid egg yolk, and liquid whole eggs [17]. Their versatility extends from household and restaurant use to serving as raw materials in various food processing applications, including meat products, mayonnaise, confectioneries, and ice cream. The liquid-egg processing typically involves washing, opening, removing shells, filtering, classification, and heat treatment [18]. During this process, the interaction between proteins and lipids can significantly influence the quality and material characteristics of the final product [19].

Previous research has demonstrated that protein–lipid interactions in foods can affect protein structure, physicochemical properties and functionality [20]. For instance, Krajewska et al. [21] observed the disappearance of OVA’s characteristic tyrosine absorption peak following interactions with stearic acid. Fang et al. [22] reported that oleic acid exposure could reveal α-lactalbumin’s tryptophan residue, leading to tertiary structure loss. Additionally, Rizzuti et al. [23] found that fatty acid isomolality enhanced β-lactoglobulin’s thermal stability. Understanding these protein–lipid interactions during liquid-egg processing is crucial for optimizing product properties.

This investigation focuses on examining the interaction between four major egg proteins (OVA, OVT, OVM, and LYZ) and four significant lipid components (phosphatidylcholine, palmitic acid, oleic acid, and linoleic acid) to elucidate their effects on protein structures and physicochemical properties during processing.

## 2. Materials and Methods

### 2.1. Materials

OVA (purity ≥ 90%), OVT (purity ≥ 90%), and LYZ (purity ≥ 90%) was prepared using anion exchange chromatography on a DEAE-Sepharose Fast Flow column (16 mm × 250 mm, Amersham Biosciences, Freiburg, Germany), following the method described by Ma [14]. The protein solution was then dialyzed against water at pH 7.0 for 48 h using membranes with a molecular weight cutoff of 10 kDa. After dialysis, the solution was lyophilized and stored at −20 °C until needed. The OVM sample with a purity ≥ 90% was obtained from Cusabio Biotech Co., Ltd. (Wuhan, China). PC and PA were supplied by Beijing Solarbio Science & Technology Co., Ltd. (Beijing China), while OA and LA were acquired from Aladdin Biochemical technology Co., Ltd. (Shanghai, China), and 8-anilino-1-naphthalenesulfonic acid was purchased from Sigma-Aldrich Chemical Co., Ltd. (St. Louis, MO, USA). All other reagents used in this study were of analytical grade.

### 2.2. Preparation of Lipids and Proteins Solution

OVA, OVT, OVM, and LYZ were dissolved in ultrapure water to achieve a final concentration of 1 mg mL^−1^, and the lipids (PC, PA, OA, and LA) were then added directly to the protein solutions in the specified egg component ratios (in proportion to the weight of each component in the egg; PC:OVA = 0.10:1, OA:OVA = 0.56:1, PA:OVA = 0.29:1, LA:OVA = 0.14:1, PC:OVT = 0.43:1, OA:OVT = 2.51:1, PA:OVT = 1.31:1, LA:OVT = 0.62:1, PC:OVM = 0.47:1, OA:OVM = 2.74:1, PA:OVM = 1.43:1, LA:OVM = 0.68:1, PC:LYZ = 1.52:1, OA:LYZ = 8.86:1, PA:LYZ = 4.64:1, LA: LYZ = 2.18:1). The solution containing the proteins and lipids was vortexed for 0.75 min, followed by incubation at 60 °C for 3.5 min. Moreover, native proteins at room temperature were used as the control (N-OVA, N-OVT, N-OVM, and N-LYZ, respectively), and other native proteins were heat-treated at 60 °C for 3.5 min (H-OVA, H-OVT, H-OVM, and H-LYZ, respectively). All samples were centrifuged for 20 min at 10,000× *g* to remove any unbound fatty acids, followed by storing at −20 °C for further use.

### 2.3. Sodium Dodecyl Sulfate–Polyacrylamide Gel Electrophoresis

The molecular weights changes in protein complexes and monomers were analyzed using non-reducing sodium dodecyl sulfate–polyacrylamide gel electrophoresis with a Phast System Electrophoresis apparatus (BIO-RAD, Hercules, CA, USA). All protein samples (1.0 mg mL^−1^) were mixed with 10 μL of non-reducing loading buffer and heated at 100 °C for 5 min. The gels were stained with Coomassie Brilliant Blue R-250 (Sigma, St. Louis, MO, USA), and the image intensity was quantified using Quantity One-4.6.2 (BIO-RAD, Hercules, CA, USA).

### 2.4. Circular Dichroism Spectrum

The secondary structural changes in all protein samples were analyzed using a MOS-450/AF-CD spectrometer (Bio-Logic, Grenoble, France) in the far-ultraviolet circular dichroism spectrum ranging from 190 to 250 nm. Spectra were recorded at 25 °C with a bandwidth of 60 nm min^−1^ and a rate of 1 nm, and all samples were diluted to 0.1 mg mL^−1^ with ultrapure water. The smoothed spectra were represented in terms of average residual molecular weight as average residual ellipticity [deg cm^2^/dmol], and the content of different secondary structures was calculated using secondary structure analysis software (http://dichroweb.cryst.bbk.ac.uk/html/home.shtml, accessed on 17 April 2023).

### 2.5. Ultraviolet Spectrum

The tertiary structure of the sample was analyzed using a TU-1901 ultraviolet-visible spectrophotometer (TU-1901, Purkinje General, Beijing, China) for ultraviolet spectroscopy. The sample was diluted with ultrapure water to a concentration of 0.2 mg mL^−1^ and analyzed within the wavelength range of 250 to 350 nm at a scanning rate of 120 nm min^−1^.

### 2.6. Fluorescence Spectrum

The surface hydrophobicity of the protein samples was assessed by measuring the fluorescence intensity in the presence of the hydrophobic fluorescent probe 8-anilino-1-naphthalenesulfonic acid. Each protein sample was supplemented with the optimal concentration of 8-anilino-1-naphthalenesulfonic acid (8 mM, 50 μL) at a final protein concentration of 0.2 mg mL^−1^ in a total volume of 5 mL. The mixtures were gently swirled and incubated for 50 min at room temperature in the dark. The fluorescence of 8-anilino-1-naphthalenesulfonic acid was excited at 390 nm, and the emission spectra were collected at a scan speed of 1200 nm min^−1^ between 400 and 650 nm.

### 2.7. Solubility

The Coomassie bright blue method [24] was used to determine the soluble protein content. The protein samples were centrifuged at 10,000× *g* at 4 °C for 20 min, and the supernatant was collected and analyzed using bovine serum protein as the standard.

The standard curve was drawn to calculate the protein content, and the formula presented as follows:

S = P_0_/P_1_ × 100%

where S is solubility (%); P_0_ is the total amount of protein in the supernatant; and P_1_ is the total amount of protein in the sample.

### 2.8. Particle Size Distribution

The particle size distribution of the protein complexes was measured by dynamic light scattering. The measured wavelength was set at 633 nm, the scattering angle was 90°, the small aperture plate was 200 μm, the test time was 5 min, and examinations of all samples were carried out in triplicate at room temperature.

### 2.9. Immunoglobulin G Binding Capacity Assessment

To analyze the antigenicity of samples, a competitive inhibition enzyme-linked immunosorbent assay was employed to detect the binding capacity of immunoglobulin G [25]. In brief, untreated proteins (OVA, OVT, OVM and LYZ) at a concentration of 1 μg mL^−1^ were pre-coated onto the wells. The competing samples included the proteins themselves, h-protein, PC-protein, OA-protein, PA-protein, and LA-protein. Rabbit polyclonal antibody (diluted 1:20,000 to ensure the binding capacity of immunoglobulin G) served as the primary antibody, while horseradish peroxidase-conjugated goat anti-rabbit immunoglobulin G (diluted 1:5000) was used as the secondary antibody. The corresponding optical density values at 450 nm were measured using a Bio-Rad 1860 microplate reader (Bio-Rad, CA, USA) to determine the immunoglobulin G binding capacity. The immunoglobulin G binding capacity for all samples was expressed as the inhibition rate, calculated using the following equation:

Inhibition (%) = (1 − B/B_0_) × 100%

where B represents the absorbance value of the wells containing the inhibitor and B_0_ represents the absorbance value of the blank control.

### 2.10. Statistical Analysis

All data were processed using GraphPad Prism 8.0 (GraphPad Software Inc., San Diego, CA, USA) and were presented as means with standard deviations unless otherwise specified. The statistical significance of the data was analyzed using analysis of variance (Tukey) procedures (SPSS, version 13.0, Chicago, IL, USA), and the significance was determined at *p* < 0.05.

## 3. Results

### 3.1. Sodium Dodecyl Sulfate–Polyacrylamide Gel Electrophoresis Analysis of the Egg Proteins

**Figure 1:**
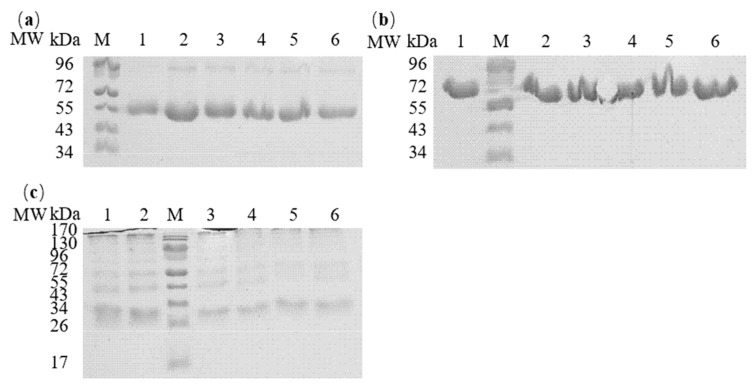
Non-reduced sodium dodecyl sulfate–polyacrylamide gel electrophoresis protein profiles of OVA (**a**), OVT (**b**), and OVM (**c**) treated with and without lipids. Lane M: Pre-stained markers. Lane 1: Native OVA, OVT, or OVM. Lane 2: h-OVA, OVT, or OVM. Lanes 3–6: PC-, PA-, OA- and LA-treated OVA, OVT, or OVM, respectively.

Polyacrylamide gel electrophoresis is a technique that separates molecules based on their sizes and charges, with various systems being available depending on the sample type and downstream applications. Sodium dodecyl sulfate–polyacrylamide gel electrophoresis, in particular, is a highly valuable method for fractionating proteins according to their molecular weight [26]. Non-reducing sodium dodecyl sulfate–polyacrylamide gel electrophoresis revealed the differential effects of lipids on protein structure under pasteurization conditions (Figure 1). For OVA (45 kDa), heat treatment induced the formation of high-molecular-weight components (~95 kDa), suggesting dimer formation (Figure 1a). While the four lipids (PC, PA, OA and LA) showed varying effects on band intensity compared to heat-treated controls, they maintained similar band patterns. In contrast, OVT, OVM, and LYZ exhibited minimal changes in their electrophoretic profiles across all treatments, indicating that these proteins maintained their primary structure integrity under the tested conditions (Figure 1b).

### 3.2. Secondary Structure Analysis of the Egg Proteins

**Figure 2:**
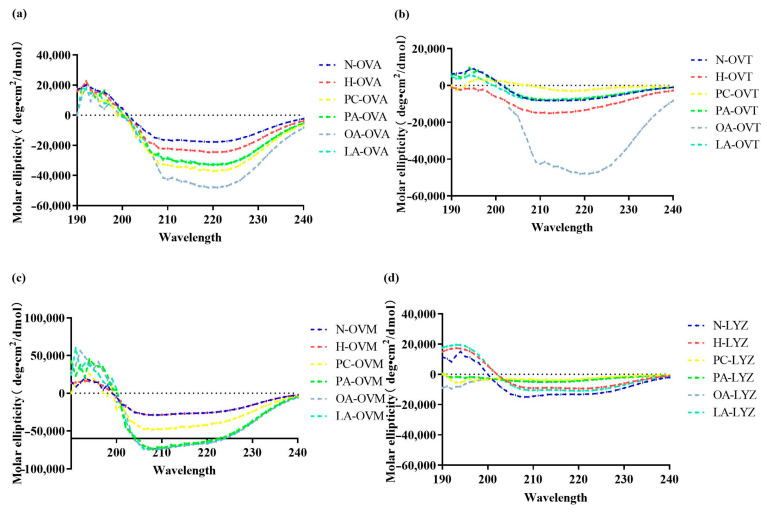
The circular dichroism spectra of OVA (**a**), OVT (**b**), OVM (**c**), and LYZ (**d**) treated with and without lipids (PC, PA, OA, and LA).

A circular dichroism spectroscopy revealed distinct lipid-dependent modifications in the secondary structures of proteins (Figure 2). OVA exhibited decreased negative molar ellipticity following heat treatment, with further reductions being found upon lipid addition, particularly at 220 nm (Figure 2a). Oleic acid demonstrated the most pronounced effect, suggesting potential covalent interactions affecting the hydrogen bonding. PC’s impact likely stemmed from its amphipathic nature, enabling both electrostatic and covalent interaction with OVA. PA and LA showed comparable effects.

In Figure 2b, OA similarly exhibited the strongest influence on secondary structures. Notably, PA, OA, and LA induced nearly identical effects on OVM at 210 nm (Figure 2c), possibly due to their shared fatty acid characteristics. LYZ displayed unique responses, with PC showing the greatest impact, likely through electrostatic interaction—a finding consistent with LYZ’s basic nature (Figure 2d).

### 3.3. Impact on Protein Tertiary Structure

**Figure 3:**
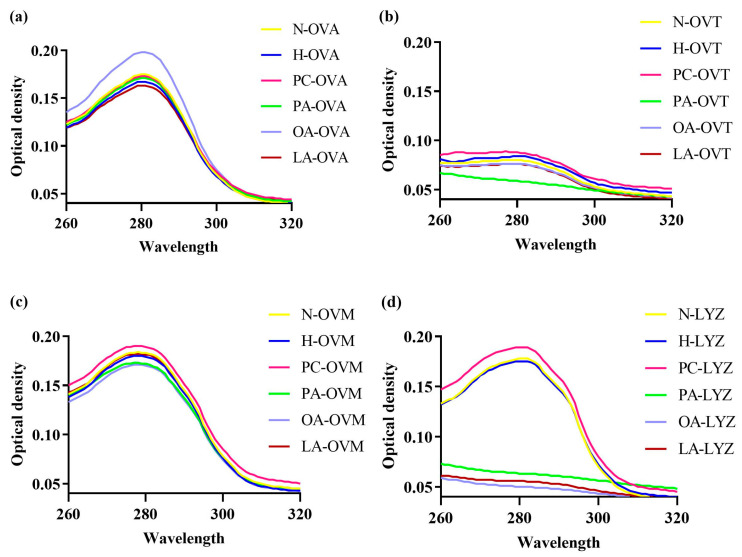
The ultraviolet spectrum spectra of OVA (**a**), OVT (**b**), OVM (**c**), and LYZ (**d**) treated with and without lipids (PC, PA, OA, and LA).

The conformational change in protein can be detected by the change in the ultraviolet absorption spectrum. The ultraviolet absorption spectrum of proteins is primarily attributed to the absorption of ultraviolet light by the side chains of tryptophan and tyrosine residues, followed by those of phenylalanine, histidine, and cysteine residues [27]. An ultraviolet spectrum spectroscopic analysis at 280 nm provided insights into changes in the protein tertiary structure (Figure 3). While mild heat treatment minimally affected OVA’s tertiary structure (Figure 3a), OA notably increased absorbance, indicating the enhanced exposure of aromatic amino acid residues. PC increased OVT’s absorption value compared to heat-treated controls, suggesting the expanded exposure of aromatic residues. Conversely, PA, OA, and LA decreased the characteristic peak absorption values for OVT, OVM, and LYZ (Figure 3b–d), indicating the burial of previously exposed residues.

### 3.4. Alterations in Surface Hydrophobicity

**Figure 4:**
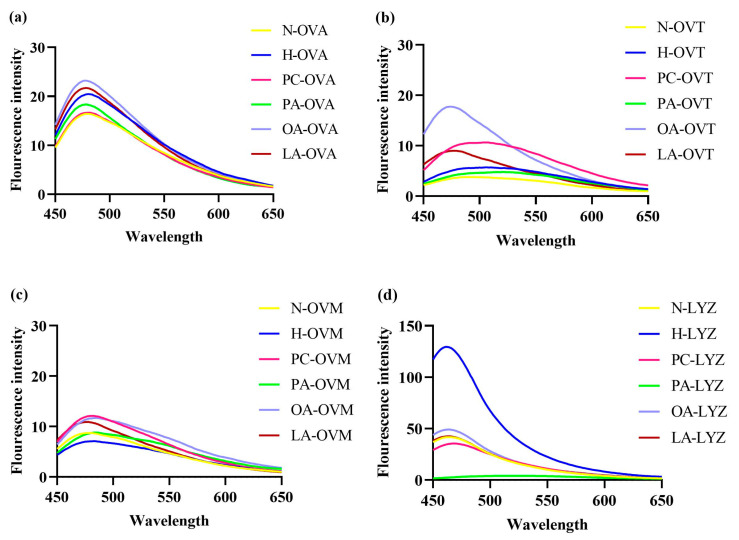
The 8-anilino-1-naphthalenesulfonic acid fluorescence spectra of OVA (**a**), OVT (**b**), OVM (**c**), and LYZ (**d**) treated with and without lipids (PC, PA, OA and LA).

Exogenous fluorescence, mainly through the fluorescence probe 8-anilino-1-naphthalenesulfonic acid, was linked to the surface hydrophobicity of proteins. Notably, 8-anilino-1-naphthalenesulfonic acid probes show a low fluorescence quantum yield in aqueous solutions. However, an increase in fluorescence can be observed when the probes bind to the exposed hydrophobic region of the protein, which is commonly used as a metric to measure protein surface hydrophobicity [28]. Additionally, 8-anilino-1-naphthalenesulfonic acid fluorescence spectroscopy revealed significant changes in protein surface hydrophobicity (Figure 4). In Figure 4a, heat-treated OVA showed an increased fluorescence intensity compared to native protein, indicating greater exposure of hydrophobic regions. OA and LA further enhanced this effect, with OA showing the strongest impact. Conversely, PA and PC decreased the fluorescence intensity below heat-treated controls, suggesting reburial of exposed hydrophobic structures.

For OVT and OVM, OA, LA, and PC increased hydrophobic exposure compared to heat-treated controls (Figure 4b,c). Notably, LYZ exhibited maximum fluorescence intensity under heat treatment alone, with all lipid additions reducing hydrophobic exposure (Figure 4d).

### 3.5. Differential Effects on Protein Solubility

**Figure 5:**
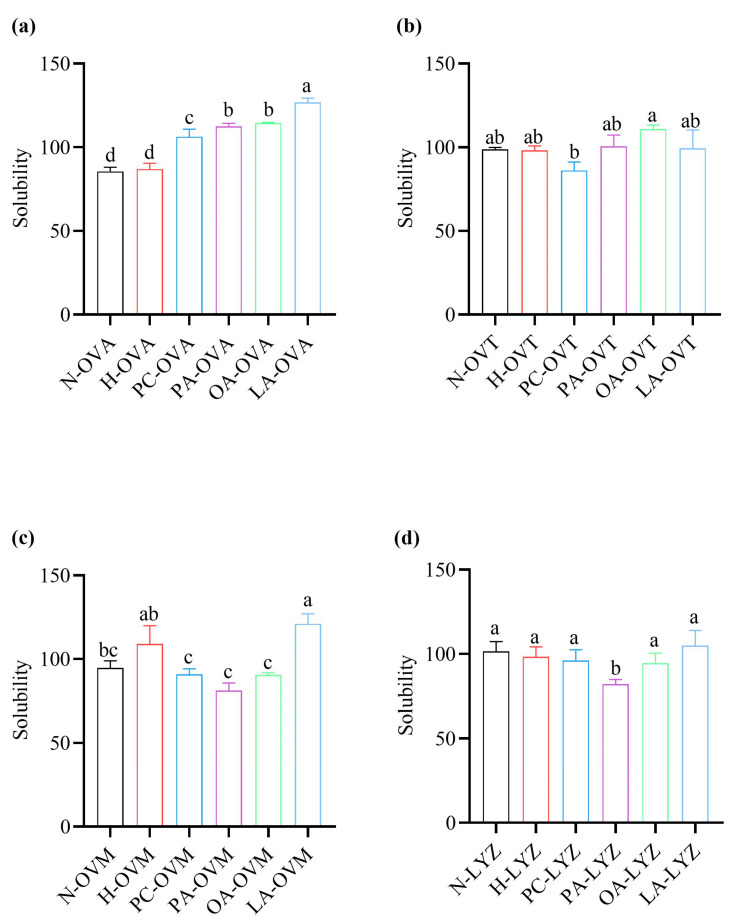
The solubility of OVA (**a**), OVT (**b**), OVM (**c**), and LYZ (**d**) treated with and without lipids (PC, PA, OA, and LA). Different letters indicate statistically significant differences between the groups (*p* < 0.05).

Solubility is a critical thermodynamic factor in the defining of protein interactions [28]. Protein solubility analyses revealed lipid-specific effects (Figure 5). While heat treatment alone maintained OVA solubility, all tested lipids enhanced it significantly, with LA showing the strongest effect. This suggests the formation of soluble protein–lipid complexes or protein–protein polymers (Figure 5a). OVT showed minimal solubility changes except for PC and OA treatments, which differed significantly from each other (Figure 5b). PC-OVM, PA-OVM, and OA-OVM exhibited decreased solubility compared to heat-treated controls, suggesting the formation of insoluble complexes (Figure 5c). LYZ maintained consistent solubility across most treatments, with only PA significantly reducing it (Figure 5d).

### 3.6. Impact on Particle Size Distribution

**Figure 6:**
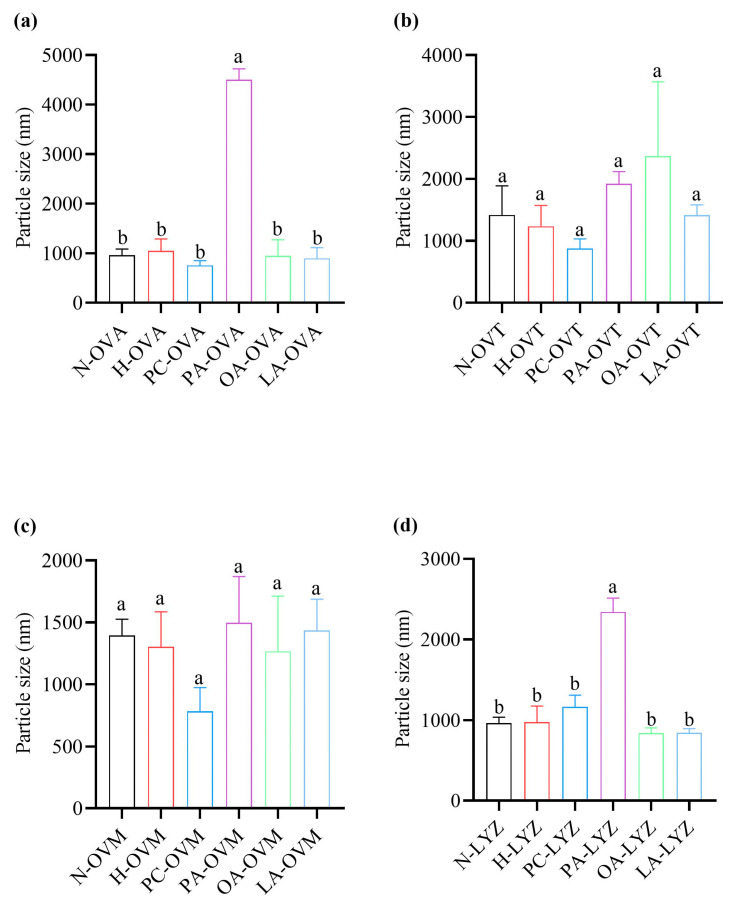
The particle sizes of OVA (**a**), OVT (**b**), OVM (**c**), and LYZ (**d**) treated with and without lipids (PC, PA, OA, and LA). Different letters indicate statistically significant differences between the groups (*p* < 0.05).

Dynamic light scattering revealed the selective effects on protein aggregation (Figure 6). PA significantly increased the particle sizes for OVA and LYZ, indicating protein aggregation or complex formation (Figure 6a,d). OVT and OVM showed no significant differences across treatments, though OA-OVT and PA-OVM exhibited the largest particle sizes within their respective groups (Figure 6b,c). Notably, the pasteurization conditions alone did not significantly affect the particle size for any protein.

### 3.7. Modulation of Protein Antigenicity

**Figure 7:**
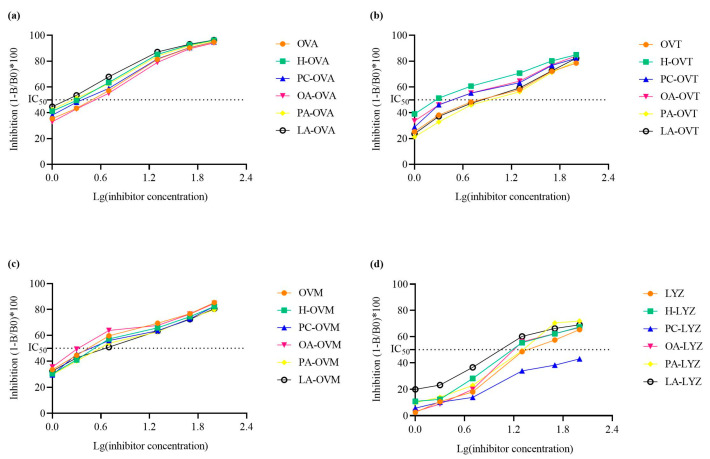
The immunoglobulin G binding capacities of OVA (**a**), OVT (**b**), OVM (**c**), and LYZ (**d**) treated with and without lipids (PC, PA, OA, and LA).

A competitive inhibition enzyme-linked immunosorbent assay demonstrated complex effects on protein antigenicity (Figure 7). OA treatment produced the highest IC_50_ value for OVA, indicating a reduction in antigenicity, while PA and LA enhanced it compared to the heat-treated controls (Figure 7a). For OVT, PA treatment showed the highest IC_50_ value, whereas OA had the most significant impact on OVM’s antigenicity (Figure 7b,c). Additionally, LA treatment resulted in the strongest antigenicity for LYZ, suggesting a strong lipid–protein interaction.

## 4. Discussion

This study provides comprehensive insights into lipid–protein interactions during egg pasteurization, a critical process in liquid egg production. Our findings reveal distinct patterns of lipid-specific effects on protein structure and functionality under pasteurization conditions (60 °C, 210 s). These insights contribute to our understanding of egg protein behavior during thermal processing and provide a foundation for optimizing liquid egg production processes and product formulations.

In this work, we found that the treatment of egg proteins with lipids in eggs caused different secondary changes. As shown in Figure 2a, the spectrum of OVA exhibits a negative band at 222 nm, indicating that the protein possesses an α-helical structure, which was consistent with the findings of Xin Qi et al. [29]. In addition, the secondary structure of OVA treated with lipids changed compared to heated OVA samples. The OA group had the lowest negative band after the addition of all lipids, which may be due to the interaction between lipids and OVA, which disrupted the ordered structure of OVA (among which OA had the highest degree) [30]. The OA group in OVT had the lowest negative band, which was similar to the result of OVA. OA and OVT had the strongest response, which meant that, compared with other groups, the structure of OA-OVT was the most disordered and there was no significant change in other groups (Figure 2b). In Figure 2c, negative bands in the lipid addition map all decreased, indicating that they may interact with OVM to make the secondary structure of OVM looser. As for Figure 2d, there was no significant change in its secondary structure.

In Figure 4a, compared to heated OVA, the fluorescence intensity of the OA and LA groups increased, while the fluorescence intensity of the PA and PC groups decreased. This may be due to the addition of the four lipids, which altered the conformation of OVA, leading to structural expansion and the exposure of internal hydrophobic amino acids [31]. Additionally, PA and PC may bind with the hydrophobic amino acids exposed by heating, resulting in a reduction in fluorescence intensity. In Figure 4b, the fluorescence intensity of heated OVT increased compared to untreated OVT, indicating an enhancement in surface hydrophobicity, which is consistent with the findings described by Wang et al. [32]. In addition, Zhang et al. [33] found that the introduction of hydrophilic groups on the surface of OVT would reduce the surface hydrophobicity, which was exactly the opposite of the results of the OA and LA groups in Figure 4b, precisely because the introduction of the hydrophobic groups by OA and LA enhanced the surface hydrophobicity of OVT. In Figure 4c, compared with the heating group, the exogenous fluorescence of the three groups (PC, OA, and LA) increased to varying degrees. This result is contrary to the research results of Rui et al. [34], which may be because the three lipids mentioned above promote the binding of 8-anilino-1-naphthalenesulfonic acid and OVM during the binding process. In Figure 4d, h-LYZ has the highest fluorescence intensity, so, under this condition, the surface hydrophobicity of lysozyme after heating is the strongest [35]. The addition of other lipids reduced the fluorescence intensity, indicating that lipids prevented the dense hydrophobic structure inside the lysozyme from being exposed to the surface.

Due to the sensitivity of the absorption spectra of aromatic amino acids to their local chemical environments, the maximum absorption wavelength and absorption intensity of proteins can be utilized to detect interactions between proteins and ligands. Generally, the absorption of proteins in the near-ultraviolet range (240–300 nm) is primarily determined by the quantity of tyrosine and tryptophan, with a smaller contribution being seen from the number of phenylalanine residues and disulfide bonds [36]. In Figure 3a, compared to the OVA after heating, only the ultraviolet spectrum-absorption intensity of OA-OVA was enhanced in the lipid group, indicating that the aromatic amino acid residues in the OVA structure were exposed to a more hydrophobic microenvironment due to their interactions with OA [37]. It can be seen from the ultraviolet spectrum absorption spectra of OVT and lipids that, compared with the heated control group, there is a lot of overlap in their spectra, so the addition of lipids has no effect on their response, and similar results are also found in OVM (Figure 4b,c) [38]. At 280 nm, the absorption peak in the protein’s ultraviolet spectrum-visible spectrum is attributed to the absorption of aromatic amino acid residues (tryptophan, tyrosine, and phenylalanine). If the microenvironment of these amino acid changes upon the incorporation of a ligand, the spectral properties of these amino acids will also be affected. In Figure 3d, the absorbance decreased after the addition of PA, OA, and LA, while the absorbance increased after the addition of PC, which indicates the change in the amino acid microenvironment and the formation of a complex between the three fatty acids and lysozyme. However, during the compound process of PC, more aromatic amino acid residues may be exposed to the microenvironment due to the amphibiality [39].

In Figure 5b, the solubility of the OA group was not significantly different from that of other groups but was significantly higher than that of the PC group. This may be due to the interaction between OA and OVT to produce soluble polymers [40]. PA increased significantly compared with the other five groups, but there was no significant difference between the other five groups in Figure 6a. This result may be attributed to the binding of PA with OVA, where the oligomerization of PA molecules promotes protein aggregation [41]. In the OVM group, there were no significant differences in the average particle size among the various groups, indicating that the interactions between the lipids and OVM have minimal amounts of electrostatic repulsion [42]. The results of OVT were similar to OVM (Figure 6b,c). The results in LYZ are similar to OVA. Therefore, it is the combination of PA and LYZ that leads to lysozyme aggregation.

Previous studies have reported that changes in the secondary and tertiary structures of proteins significantly affect their immunoglobulin G binding capacity and are directly related to epitopes, including conformational and linear epitopes [43]. Ultraviolet spectrum absorbance, exogenous fluorescence, and circular dichroism spectra show that the tertiary structure of OVA samples treated with OA develops through interaction, and the secondary structure also changes. Figure 7a shows that the antigenicity of OA-OVA is the weakest, possibly because some epitopes that were originally exposed were buried again [44]. In Figure 7b, the antigenicity of OA decreased compared with the heated OVT, and structural analysis proved that the hydrophobicity and secondary structure of OA-OVT were changed, so it may be because more epitopes were buried inside the molecule as a result of the unfolding of proteins [45].

It is worth noting that the antigenicity of OVM after heating is lower; then, the addition of OA and PC increases the antigenicity, which may be because the unfolding of proteins leads to more antigen epitope exposure, while the antigenicity of LA and PA is weakened for similar reasons to OVA and OVT (Figure 7a–c) [44]. In addition, the antigenicity of the lysozyme group of LA was enhanced while other lipids were weakened (Figure 7d).

## 5. Conclusions

This observation suggests a potential correlation between the unfolding of hydrophobic structures induced by OA and the observed outcome while indicating that oleic acid has a pronounced influence on the OVA structure and immunoglobulin G binding capacity. Therefore, in our daily life, we should avoid adding oleic acid to egg or egg white in the pasteurization process, which may aggravate the allergy symptoms of egg albumin patients or egg allergy patients. In addition, the addition of oleic acid will also lead to the structural deterioration of egg albumin and egg white liquid, as well as the decline of egg liquid material properties.

## Data Availability

The original contributions presented in this study are included in the article. Further inquiries can be directed to the corresponding author.
